# 

**Published:** 1908-02-22

**Authors:** 


CONTENTS.

| | PAGE |
|---|---|
| **The Experimental Transmission of Cancer...** | 539 |
| **Surgery and the General Practitioner** ... | 540 |
| **Annotations**—Anæsthetic Fatalities—Serum Treatment—The Colouring of Milk | 541 |
| **Medical Opinion and Movement** | 542 |
| **Hospital Clinics**—Intra-thoracic New Growths. By Frederick Taylor, M.D., F.R.C.P. ... | 545 |
| **Illustrative Cases**—Spinal Hemiplegia | 547 |
| **Points in Treatment**—Anti-typhoid Vaccine | 548 |
| **Points in Surgery**—Rodent Ulcer | 549 |
| Some Diseases of the Male Genital System | 550 |
| **Notes on Current Neurology**—The Cortical Centre for Hearing | 551 |
| **Dermatology** | 551 |
| **Therapeutics**—Prescriptions and Dispensing ... | 552 |
| **Public Health and Hygiene**—Details of Medical Inspection ... | 553 |
| **Resident Medical Officers' Department**—The R.M.O. and the County Court | 554 |
| **"The Hospital" Medical Book Supplement.—No. IV.** ... | 555 |
| **Hospital Administration**—Graduate Study in London | 559 |
| News and Coming Events | 561 |
| **Nursing Administration**—The Common Task... | 563 |
| **Editor's Letter-Box** ... | 563 |
| **The Graduates' Medical Schools** ... | 564 |

